# Accuracy of the Hounsfield Unit Values Measured by Implant Planning Software

**DOI:** 10.3390/dj12120413

**Published:** 2024-12-17

**Authors:** Koudai Nagata, Yusuke Kouzai, Keitaro Inaba, Manabu Fujii, Mihoko Atsumi, Katsuhiko Kimoto, Shinji Kuroda, Hiromasa Kawana

**Affiliations:** 1 Department of Regenerative Implant Dentistry, Kanagawa Dental University, Yokosuka 238-8580, Japan; 2 Department of Education Planning, Kanagawa Dental University, Yokosuka 238-858, Japan; 3 Department of Diagnostic Imaging, Kanagawa Dental University, Yokosuka 238-8580, Japan; 4 Department of Oral Microbiology, Kanagawa Dental University, Yokosuka 238-8580, Japan; 5 Department of Fixed Prosthodontics, Kanagawa Dental University, Yokosuka 238-8580, Japan; 6 Department of Oral and Maxillofacial Implantology, Kanagawa Dental University, Yokosuka 238-8580, Japan

**Keywords:** cone–beam computed tomography (CBCT), dental implant, implant planning software, multidetector row computed tomography (MDCT), Hounsfield units (HUs)

## Abstract

**Background**: The measurement of Hounsfield units (HU) during implant treatment planning is important. Currently, various manufacturers’ implant planning software programs offer HU capabilities; however, their accuracy remains unverified. In this study, we aimed to validate the accuracy of HU values measured by implant planning software programs. **Methods**: This study used one type of multidetector computed tomography (MDCT), two types of cone–beam computed tomography (CBCT), and four implant planning software packages. Three specimens were prepared for the evaluation of HUs, and the standard values of the HUs were measured. Digital Imaging and Communications in Medicine (DICOM) data obtained from MDCT and CBCT were loaded into four implant planning software packages to measure the HU values. The HU and reference values of the four implant planning software programs obtained from MDCT and CBCT were compared. Additionally, the HU values between each implant planning software program were compared. **Results**: The HU values of the three specimens, as measured using the four implant planning software programs utilizing MDCT, did not exhibit a significant difference from the standard values. Conversely, those obtained from CBCT were significantly different. The measured HU values after the MDCT imaging of the specimens were not significantly different between the implant planning software programs; however, they differed after CBCT imaging. **Conclusions**: The results of this study indicate that it is not possible to measure HU values using CBCT with implant planning software programs. However, HU values can be measured by any implant planning software using MDCT.

## 1. Introduction

Radiographs serve an important role in dental care. Panoramic radiographs provide an overall view of the oral cavity; however, they do not allow three-dimensional bone evaluation [1,2]. Therefore, multidetector row computed tomography (MDCT) is performed as a preoperative examination for implant treatment to evaluate the anatomy of the maxillofacial region. Cone–beam computed tomography (CBCT) has also been widely adopted for this purpose [3]. MDCT uses air and water as reference values and measures the voxel value of the target to measure the Hounsfield units (HUs), facilitating the evaluation of bone quality [4]. The National Institutes of Health defines bone quality as “the sum of all characteristics of bone that influence the bone’s resistance to fracture”. Bone quality comprises bone structure, metabolism, and mineralization, as well as the accumulation of microdamage [5].

It is critical to assess a patient’s HU values in implant treatment because good bone quality is significantly related to implant stability and improves the success rate of implant surgery [6,7]. For implant surgery, bone quality can be classified based on HU values [8,9]. Cavdar et al. performed socket–socket preservation at 52 maxillary anterior tooth sites, measured the HU values via MDCT after 120 days, and highlighted its utility in determining the optimal timing for implant placement [10]. Khaled et al. measured the HU values after sinus elevation to assess bone quality and determine the timing of implant placement [11]. However, MDCT has a high radiation dose, and capturing multiple MDCT images during implant treatment raises ethical issues [12]. MDCT is equipped with a filter to remove scattered rays, whereas CBCT is not and is, therefore, susceptible to the effects of scattered rays. Furthermore, the field of view (FOV) of CBCT is small, and true CT values cannot be obtained because not all projection data of the patient are available, and image reconstruction is performed from the incomplete projection data [13]. Pauwels et al. reported that the inability to measure HU values with CBCT was attributed to high scattering, limitations in imaging range, and reconstruction algorithms [14]. However, many dental clinics use CBCT for implant treatment, as it has lower radiation exposure, higher resolution, and lower cost than MDCT. Therefore, challenges have been raised with the use of gray values obtained from CBCT as pseudo-HU values in bone quality assessment [15]. Selvaraj et al. conducted a study to assess bone mineral density by examining the correlation between gray values obtained from CBCT and HU values obtained from MDCT; they analyzed 14 studies and reported a moderate positive correlation (r = 0.669), albeit statistically insignificant [16]. However, a systematic review by Eguren et al. reported that the gray values obtained from CBCT cannot currently be converted to HU due to insufficient clinical studies with diagnostic capability [17].

Advancements in digital technology have facilitated the utilization of Digital Imaging and Communications in Medicine (DICOM) data in implant planning software after CT imaging, aiding in implant placement site determination, surgical guide creation, and dynamic navigation during surgery [18,19]. Currently, many companies have produced implant planning software to support surgeons in performing implant treatment, and each software is equipped with a system to measure the HU values. Al-Ekrish tested the accuracy of three different implant planning software programs and found no significant differences in their error values [20]. Despite reports on software accuracy, the validation of HU measurement tools within implant planning software remains unexplored, thus leaving the accuracy of these features unknown. As mentioned previously, HU measurement affects the success rate of implant treatment and determines the timing of implant placement after socket preservation and sinus elevation. In this study, our aim was to validate the accuracy of HU measurement tools installed in implant planning software. This study used one type of MDCT, two types of CBCT, and four implant planning software packages. We hypothesized that there would be no difference in the HU measured by the four types of implant planning software after MDCT and CBCT imaging.

## 2. Materials and Methods

### 2.1. Specimen Preparation

**Figure 1:**
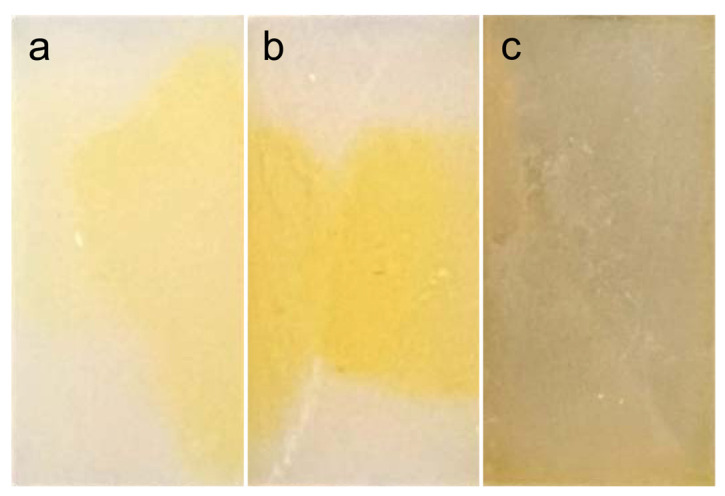
Three specimens with different hydroxyapatite contents were prepared. (**a**) Hydroxyapatite content of 200 mg/cm^3^ was set as Specimen 1. (**b**) Hydroxyapatite content of 100 mg/cm^3^ was set as Specimen 2. (**c**) Hydroxyapatite content of 0 mg/cm^3^ was set as Specimen 3.

A block-type phantom, the Bone Mineral Density Chart Phantom (UHA, Kyoto Kagaku, Kyoto, Japan), was used to measure the HUs in the implant planning software. One characteristic of this specimen is its even distribution of hydroxyapatite. Therefore, we selected this specimen for accurate evaluation of HUs. Three specimens were prepared with hydroxyapatite contents of 200 mg/cm^3^, 100 mg/cm^3^, and 0 mg/cm^3^ for HU value evaluation (Figure 1) and were designated as Specimens 1, 2, and 3, respectively.

### 2.2. CT Imaging

One type of MDCT and two types of CBCT were used to perform imaging on the specimens. MDCT was performed using Aquilion PRIME (©CANON MEDICAL SYSTEMS CORPORATION, Tochigi, Japan). The imaging conditions for the MDCT and CBCT devices utilized in this study were as follows: For MDCT: FOV, φ24 × H24; tube current, 50 mA; tube voltage, 120 kVP; slice thickness, 0.5 mm; and voxel size, 0.05 mm.

One of the CBCT devices used was OP 3D Vision (EH Japan Co., Ltd., Tokyo, Japan). The imaging conditions were as follows: FOV, φ8 × H8; tube current, 50 mA; tube voltage, 120 kVP; slice thickness, 0.5 mm; and voxel size, 0.02 mm.

The second CBCT was performed using a 3DX device (Morita Co. Ltd., Tokyo, Japan). The imaging conditions were as follows: FOV, φ10 × H10; tube current, 5 mA; tube voltage, 89 kVP; slice thickness, 0.5 mm; and voxel size, 0.02 mm. Following this description, the CBCT conducted with the OP 3D Vision device was denoted as CBCT1, while the CBCT performed with the 3DX device was denoted as CBCT2. Throughout the study, the specimen was positioned centrally within the FOV and aligned perpendicular to the cross-section (axial) of the X-ray beam.

### 2.3. HU Measurement Method

**Figure 2:**
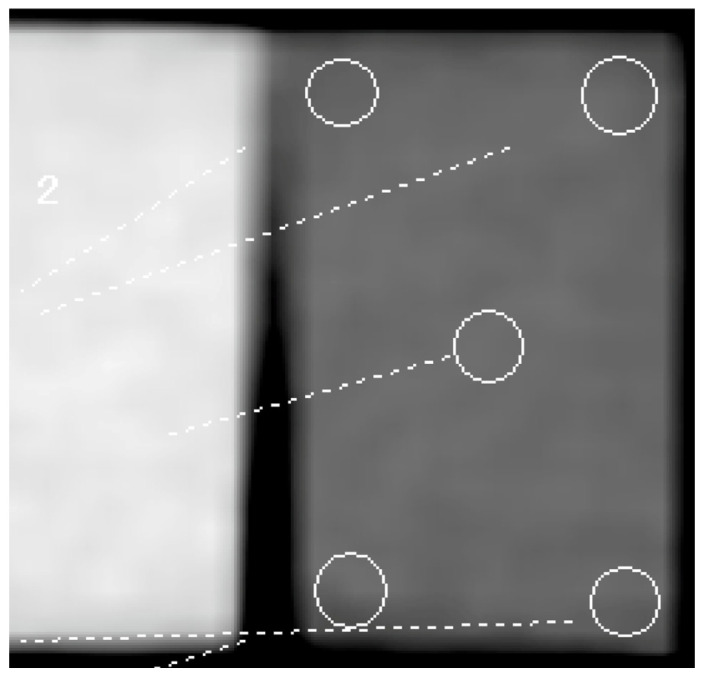
Determination of standard values. Three specimens were initially imaged with MDCT, and the HU values were measured using SYNAPSE Result Manage. Measurements were taken in the same plane, with the HU values measured at the center and four corners, and the average value was used as the result. MDCT, multidetector computed tomography; HUs, Hounsfield units.

**Figure 3:**
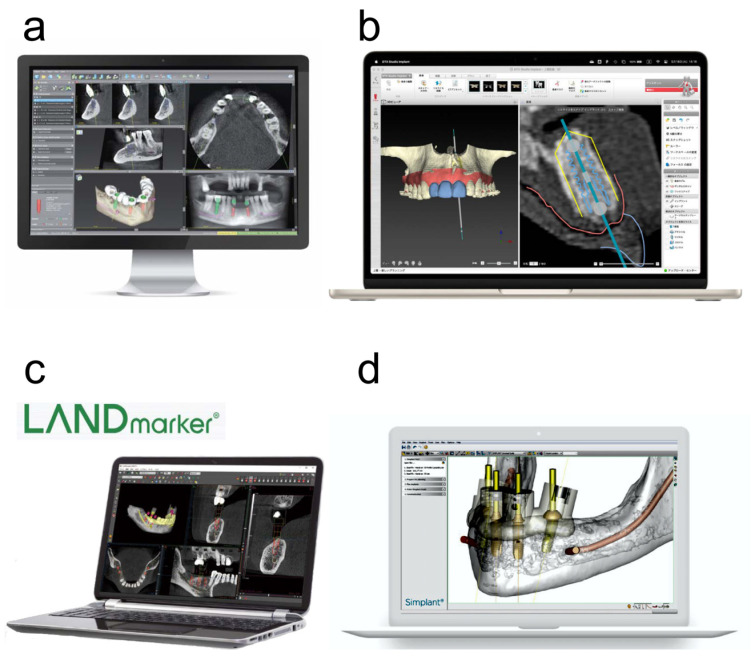
Four implant planning software programs were used. (**a**) coDiagnostiX^®^. (**b**) DTX Studio™ Implant. (**c**) LANDmarker^®^. (**d**) Simplant.

**Figure 4:**
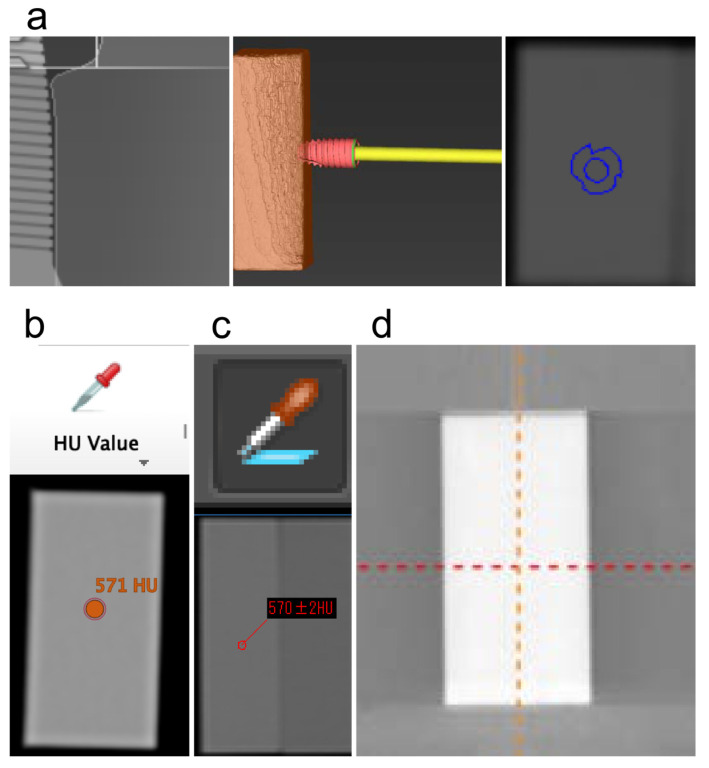
Methods of measuring the HU values differed for each implant planning software program. (**a**) HU values are measured using co by placing the tip of a simulated implant into the specimen. (**b**) In DTX, HU values are measured by selecting the HU value measurement tool at an arbitrary site. (**c**) In LAND, HU values are measured by selecting the HU value measurement tool at any site. (**d**) In Sim, HU values are measured by positioning the cursor on an arbitrary area. The measurement site was the same surface as that of the standard values; the HU values were measured at the center and four corners, and the average values were obtained. HUs, Hounsfield units.

To determine the reference values, the three specimens were first imaged using MDCT. Subsequently, HU measurements were conducted using the Picture Archiving and Communication System (SYNAPSE Result Manager version 3, FUJIFILM, Tokyo, Japan) with soft tissue display mode images. The specimens were measured on the same surface, in the center, and at the four corners. The HU values were measured, and the average value was used as the result (Figure 2). Next, the standard value (SV) for each specimen was set. Then, CBCT imaging was performed on the specimens, and DICOM data obtained from MDCT and CBCT were used to measure the HU values using four different implant planning software packages. As each implant planning software determines the HU value by referencing the gray value of the DICOM data obtained from the CBCT imaging, the HU value was measured, not the gray value. The first implant planning software utilized in this study was coDiagnostiX^®^ version 9.19 (Dental Wings, Montreal, Canada), hereafter abbreviated as “co”; the second was DTX Studio™ Implant version 3.6.8.1 (Nobel Biocare AG, Kloten, Switzerland), hereafter referred to as “DTX”; the third was LANDmarker^®^ version 8.04 (iLAND solutions Co., Ltd., Osaka, Japan), hereafter referred to as “LAND”; and the fourth was Simplant version 16 (Dentsply Sirona K.K., Tokyo, Japan), hereafter referred to as “Sim” (Figure 3). As co can measure the HU values by simulating the implant, the measurement was performed by placing the tip of the simulated implant body against the specimen. DTX and LAND can measure the HU values by selecting a HU measurement tool for any site. Sim can also measure the HU values by hovering the cursor over any site (Figure 4). Measurements were consistently recorded in the same plane as the reference value. The HU value at each site was measured uniformly, with readings taken at the center and at the four corners; subsequently, the results were averaged. All HU values for each implant planning software program were automatically measured according to the manufacturer’s specified protocol.

### 2.4. Study Items

In this study, four items were established to evaluate the accuracy of the HU value using implant planning software.

Study Item 1: For the SV of each specimen, we verified whether there was any difference in the HU values measured using the four implant planning software programs, derived from the DICOM data obtained post-MDCT imaging.

Study Item 2: Following MDCT imaging of each specimen, we examined whether there were any differences in the HU values measured using the four implant planning software programs, based on the acquired DICOM data.

Study Item 3: For each specimen’s SV, we examined whether differences existed in the HU values measured by the four implant planning software programs, utilizing DICOM data obtained after CBCT imaging conducted by the two machines.

Study Item 4: After each specimen was imaged by the two CBCT scans, we verified whether there was any difference in HU values between the four implant planning software programs, based on the acquired DICOM data.

### 2.5. Statistical Analyses

For test items 1 and 3, a Dunnett’s test was performed on the SV of each sample using a two-tailed test after one-way ANOVA using Microsoft Excel version 2411 (Microsoft Corp., Redmond, WA, USA). Study items 2 and 4 were verified using the Tukey–Kramer method with a two-tailed test after one-way ANOVA, as the variances of each group were equal, and the distribution was normal. The level of significance was set at *p* < 0.05. The sample size was not measured, as this was a model study.

## 3. Results

### 3.1. Study Item 1

For specimen 1, SV was 287.6 ± 6.9, co was 273.4 ± 14.4, DTX was 293.8 ± 6.9, LAND was 279.8 ± 7.9, and Sim was 291.8 ± 4.1. In specimen 2, SV, co, DTX, LAND, and Sim were 142.2 ± 7.1, 132.2 ± 11.8, 145.6 ± 3.7, 136.2 ± 6.0, and 142 ± 3.2, respectively. In specimen 3, SV was −29.4 ± 4.6, co was −36 ± 3.2, DTX was −24.6 ± 3.8, LAND was −30.4 ± 6.8, and Sim was −25 ± 3.7.

**Figure 5:**
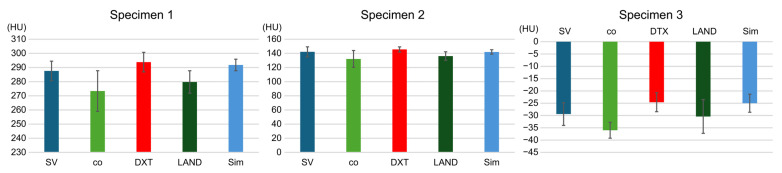
After MDCT imaging, the HU values measured by implant planning software are not significantly different compared to the SV of the three specimens. HUs, Hounsfield units; MDCT, multidetector computed tomography.

Following MDCT imaging, the HU values measured by the implant planning software were not significantly different compared to the SV of the three specimens (Figure 5).

### 3.2. Study Item 2

For specimen 1, co was 273.4 ± 14.4, DTX was 293.8 ± 6.9, LAND was 279.8 ± 7.9, and Sim was 291.8 ± 4.1. In specimen 2, co, DTX, LAND, and Sim were 132.2 ± 11.8, 145.6 ± 3.7, 136.2 ± 6.0, and 142 ± 3.2, respectively. In specimen 3, co was −36 ± 3.2, DTX was −24.6 ± 3.8, LAND was −30.4 ± 6.8, and Sim was −25 ± 3.7.

**Figure 6:**
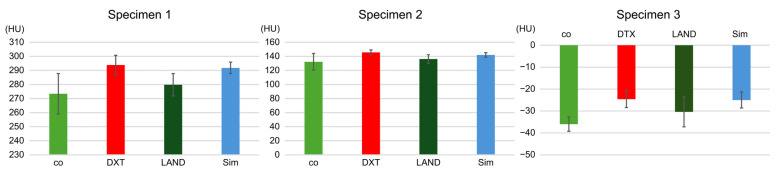
After imaging with MDCT, the measured HU values of the three specimens are not significantly different between all implant planning software programs. HUs, Hounsfield units; MDCT, multidetector computed tomography.

Following MDCT imaging, the measured HU values of the three specimens were not significantly different between all the implant planning software programs (Figure 6).

### 3.3. Study Item 3

The HU values of the specimens measured using the four implant planning software programs after imaging with the two CBCT devices showed significant differences in all cases concerning the SV of the specimens (*p* < 0.001) (Table 1).

### 3.4. Study Item 4

In the CBCT1-measured group, significant differences were noted between the values obtained from co and DTX, co and LAND, and co and Sim in Specimen 1 (*p* < 0.05) and between DTX and LAND in Specimen 3 (*p* < 0.05).

**Figure 7:**
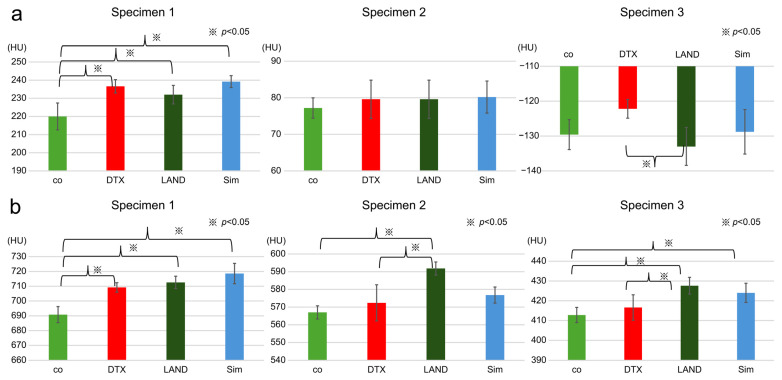
(**a**) In the group measured by CBCT1, there were significant differences between co and DTX, co and LAND, and co and Sim in Specimen 1 (*p* < 0.05) and between DTX and LAND in Specimen 3 (*p* < 0.05). (**b**) In the CBCT2-measured group, there were significant differences in co and DTX, co and LAND, and co and Sim in Specimen 1 (*p* < 0.001). Significant differences were also observed between co and LAND, and DTX and LAND in Specimen 2, and between co and LAND, co and Sim, and DTX and LAND in Specimen 3. CBCT, cone–beam computed tomography.

In the CBCT2-measured group, significant differences were observed in the values obtained from co and DTX, co and LAND, and co and Sim in Specimen 1 (*p* < 0.001). Significant differences were also observed between co and LAND, and DTX and LAND in Specimen 2, and co and LAND, co and Sim, and DTX and LAND in Specimen 3 (Figure 7).

## 4. Discussion

The results of this study showed that the HU value measured using implant planning software programs following MDCT imaging was not significantly different from the SV. Moreover, there were no significant differences observed among the implant planning software programs, implying that any of them could be effectively utilized for measuring HU values and assessing bone quality when MDCT imaging is employed. However, all the HU values measured using the implant planning software after CBCT imaging significantly differed from the SV of each specimen. Additionally, considerable errors were evident in the HU values measured with both CBCT1 and CBCT2. Therefore, our hypothesis was rejected.

Our findings suggest that the HU values measured by CBCT cannot reliably be used for evaluating bone quality using any implant planning software. Cheng et al. reported that, among 15 scanners imaging the same phantom, 14 exhibited HU variations within one standard deviation of the mean. They concluded that a consistent HU value could be obtained irrespective of the type of MDCT used [21]. MDCT can accurately calibrate X-ray attenuation values on a standard HU scale based on reference values of −1000 HU for air, 0 HU for pure water, and +1000 HU for cortical bone, thereby facilitating accurate HU measurements [22]. Therefore, there were no significant differences between Study Items 1 and 2.

Following CBCT imaging, numerous validations have been performed to determine whether gray values can serve as substitutes for HUs [23,24]. Pauwels et al. scanned a phantom with 13 CBCTs and 1 MDCT to measure HUs. They observed a moderate to high correlation between gray values obtained from CBCT and HU values obtained from MDCT. However, they noted that the average error of voxel values on CBCT ranged from 35 to 1562, posing challenges in accurately measuring HU values from CBCT [25]. Razi et al. compared HU values obtained from MDCT and gray values obtained from CBCT in the soft and hard tissues of 21 patients. They reported that both soft and hard tissues exhibited a high correlation, yet significant differences were observed below *p* < 0.001 [26]. Our study item 3 also showed a large error between the SV taken by MDCT and the HU measured by CBCT1 and CBCT2. Our results are similar to those of Razi et al.’s report and suggest the unreliability of HU values obtained from CBCT. Many factors are believed to have an influence on the gray values of CBCT images [27]. Rodrigues et al. created a phantom consisting of three cylinders filled with distilled water, plaster, and motor oil and performed CBCT imaging in three FOV sizes to assess gray values. They reported significant differences in all phantoms across each FOV [28]. Shokri et al. also examined the gray values obtained from CBCT using two different FOVs across three phantoms, reporting significantly higher values for the larger FOV. Cerabone exhibited FOV values of 1107.6 for the small FOV and 1610.6 for the large FOV, suggesting a correlation between FOV size and gray value, wherein larger FOVs yielded higher gray values [29]. In this study, CBCT1 had a FOV of φ8 × H8, while CBCT2 had a FOV of φ10 × H10. Therefore, although different CBCT models were used for the imaging, the HU values were larger for CBCT2 with a larger FOV. Thus, the FOV size can affect the voxel value. Yadegari et al. believed that the region of interest (ROI) location is responsible for the error between the gray values obtained from CBCT and the HU values obtained from MDCT. In a comparison of gray and HU values, they reported that the anterior mandibular ROI had the smallest error, whereas the posterior mandibular teeth showed the largest error, with a gray value of 1925.3 and a HU value of 1535.2; all measurement points were significantly different. The slice thickness and measurement site may have a significant impact on the measurement of gray values [30]. In both CBCT1 and CBCT2, there was a significant difference between the values obtained from co and the other three implant planning software programs in Specimen 1. Especially, co measured the HU value by simulating the implant body, which differed significantly from the measurement methods of the other three implant planning software programs, as it could measure the HU value by simulating the implant.

For DTX, Land, and Sim, the HU of the test specimen’s surface could be measured by placing the cursor. However, for co, the simulated implant was brought into contact with the test specimen to measure the HU. Therefore, we believe that co may not have been able to measure the surface of the specimen as accurately as the other three types, and this is why we found different significant differences between the respective planning software in study item 4. In DICOM data obtained from CBCT, slice thickness is important for determining voxel values because of the reconstruction of radiographic data [31]. Slice thickness is a factor that determines the voxel values. Each implant planning software assigns voxel values of slices as HU values by referring to shading by each manufacturer; therefore, the setting method differs among manufacturers. In addition, CBCT2 has a larger irradiation field and may have produced more scattered radiation than CBCT1. Increased scattered rays result in decreased contrast and increased noise. We believe that the results of study item 3 differed because of the differences in the imaging model and specimen. The HU value depends on the scanner type, tube voltage, FOV, reconstruction algorithms, including artifact reduction, and processing filters [32]. Therefore, it is impossible to measure HU values with CBCT; however, equipment calibration, prediction equation models, and a standard formula (converting gray scale values to HUs) are important to obtain pseudo-HUs. According to a systematic review by Eguren et al., to measure HUs with CBCT, the scanner must be calibrated for X-ray absorption in air and water. To our knowledge, only three reports have been published on using the correlation, prediction model, and conversion formula required for this, and they report that there is currently no scientific evidence to measure pseudo-HUs [17]. Pyakurel et al. compared multisource CBCT (ms-CBCT) with conventional CBCT and MDCT imaging after converting HUs and gray scale values to bone mineral density using scanner-specific calibration functions. The discrepancies between CBCT and MDCT were 23–27% at 90 kVp and 18–19% at 110 kVp, while the discrepancies between ms-CBCT and MDCT were significantly reduced to 10% at 90 kVp and 6% at 110 kVp, according to the report. The cone angle of the ms-CBCT beam was 2.3°, while that of the CBCT beam was less than 10°, suggesting that the smaller cone angle reduced scattering and improved the uniformity and accuracy of the CT HU values [33]. Thus, although various methods for measuring HUs using CBCT have been reported, no definitive method has been established.

Currently, various socket preservation and sinus elevation materials are available for successful implant treatment [34,35,36]. To assess bone quality after bone replacement for successful implant treatment, several studies have reported measuring gray values and converting them to HUs after CBCT. Tabrizi et al. reported the HU value at 9 months after sinus elevation to be 237.20 ± 55.72 HU in the mineralized bone group and 634.8 ± 166.11 HU in the deproteinized bone group [37]. Loveless et al. also compared HU values in groups with and without bone replacement following tooth extraction. They reported that the HU value of the alveolar bone without bone replacement was 54.7, whereas that of the group with bone replacement was 235.3 [38]. Although it is acceptable to use only the gray values obtained from CBCT images as reference values, we believe that using them as HU values during treatment planning should be avoided. Inaccurate HU measurement methods can lead to errors in determining implant placement timing and poor implant stability due to poor primary stability. Socket preservation and the decision of when to place implants after maxillary sinus floor elevation often depend on the surgeon’s experience [39]. In a report that measured the rate at which bone replacement materials were replaced with new bone, the new bone volumes of NEOBONE, Bio-Oss, and Cytrans were reported to be 4.7, 39.5, and 75.2%, respectively, at 12 weeks postoperatively [40]. Although this replacement rate has been reported, it is unclear what percentage of new bone is sufficient for implant placement. Misch and others defined the HUs for implant placement as 150 or more [8]; however, new bone grafting materials have been developed in recent years, and the HUs for these have not been measured. We believe that accurate HU measurement will help determine the timing of implantation and shorten treatment time. Jeong et al. conducted a comparative analysis of effective radiation doses for mandibular imaging using MDCT and CBCT. Their findings revealed the optimal standard-dose MDCT to be 425.84 µSv, low-dose MDCT to be 199.38 µSv, standard-dose CBCT to be 111.6 µSv, and low-dose CDCT to be 83.09 µSv [41]. Therefore, to accurately measure HUs, it is desirable to use low-dose MDCT to reduce radiation exposure and measure HUs only to determine the timing of implant insertion after bone augmentation. In future research, we intend to use MDCT to follow the changes in HUs over time after bone grafting to clarify these matters.

A limitation of this study is the limited number of imaging devices and specifications, as our university-affiliated hospital owns only two CBCTs. In addition, the irradiation field is defined by each company, which makes it difficult to ensure consistency. Increasing the number of imaging models and matching conditions for CBCT and reducing scattered radiation by reducing the irradiation field and cone angle may allow for a better understanding of pseudo-HUs. Furthermore, the HUs of bone are 1000, which differ significantly from the HUs of the specimens used in the present study.

## 5. Conclusions

While it is not possible to measure HU values using CBCT with implant planning software programs, HU measurement from MDCT images remains viable with any implant planning software. Therefore, accurate HU measurements using low-dose MDCT imaging, aimed at reducing radiation exposure, may lead to the determination of the optimal timing of implant placement following bone augmentation. Our findings establish a theoretical foundation for advancing our approach, which may streamline treatment procedures and reduce treatment duration.

## Figures and Tables

**Table 1:** Results of study item 3.

		CBCT1				CBCT2			
Specimen 1	SV	co	DTX	LAND	Sim	co	DTX	LAND	Sim
Mean	287.6	220	236.6	232	239.2	690.8	709.2	712.6	718.6
Standard deviation	6.9	7.4	3.7	5.1	3.3	5.5	3.2	4.2	6.9
Significant difference		*p* < 0.001	*p* < 0.001	*p* < 0.001	*p* < 0.001	*p* < 0.001	*p* < 0.001	*p* < 0.001	*p* < 0.001
**Specimen 2**									
Mean	142.2	77.2	79.6	79.6	80.2	567	572.4	591.8	576.8
Standard deviation	7.1	2.8	5.2	5.2	4.4	3.7	10.2	3.7	4.6
Significant difference		*p* < 0.001	*p* < 0.001	*p* < 0.001	*p* < 0.001	*p* < 0.001	*p* < 0.001	*p* < 0.001	*p* < 0.001
**Specimen 3**									
Mean	−29.4	−129.6	−122.2	−133	−128.8	412.8	416.6	427.6	424
Standard deviation	4.6	4.3	2.7	5.4	0.4	3.8	6.4	4.2	4.9
Significant difference		*p* < 0.001	*p* < 0.001	*p* < 0.001	*p* < 0.001	*p* < 0.001	*p* < 0.001	*p* < 0.001	*p* < 0.001

CBCT, cone–beam computed tomography.

## Data Availability

The data presented in this study are available upon request from the corresponding author due to ethical restrictions.
